# RNA Virus Gene Signatures Detected in Patients With Cardiomyopathy After Chemotherapy; A Pilot Study

**DOI:** 10.3389/fcvm.2022.821162

**Published:** 2022-03-11

**Authors:** Kyle Varkoly, Shaoyuan Tan, Roxana Beladi, David Fonseca, Isabela Rivabem Zanetti, Simona Kraberger, Chintan Shah, Jordan R. Yaron, Liqiang Zhang, Michael Juby, Ayman Fath, Sriram Ambadapadi, Melanie House, Paul Maranian, Carl J. Pepine, Arvind Varsani, Jan Moreb, Stacey Schultz-Cherry, Alexandra R. Lucas

**Affiliations:** ^1^Department of Internal Medicine, McLaren Macomb Hospital- Michigan State University College of Human Medicine, Mt Clemens, MI, United States; ^2^Center for Personalized Diagnostics, The Biodesign Institute, Arizona State University, Tempe, AZ, United States; ^3^Department of Infectious Diseases, St. Jude Children's Research Hospital, Memphis, TN, United States; ^4^Department of Neurological Surgery, Ascension Providence Hospital- Michigan State University College of Human Medicine, Southfield, MI, United States; ^5^The Biodesign Center of Fundamental and Applied Microbiomics, Center for Evolution and Medicine, School of Life Sciences, Arizona State University, Tempe, AZ, United States; ^6^Division of Hematology/Oncology, Department of Medicine, University of Florida, Gainesville, FL, United States; ^7^School for Engineering of Matter, Transport and Energy, Ira A. Fulton Schools of Engineering, Arizona State University, Tempe, AZ, United States; ^8^Department of Internal Medicine, Dignity Health, Creighton University, Phoenix, AZ, United States; ^9^Division of Cardiology, Department of Medicine, University of Florida, Gainesville, FL, United States; ^10^Hematologic Malignancies, Transplantation and Cellular Therapy Program, Forsyth Medical Center, Derrick L Davis Cancer Center, Winston-Salem, NC, United States; ^11^Center for Immunotherapy, Vaccines and Virotherapy, The Biodesign Institute, Arizona State University, Tempe, AZ, United States

**Keywords:** virus, infection, RNA, immune suppression, chemotherapy, LVEF, cardiomyopathy, cancer

## Abstract

**Background:**

Viral infections are pervasive and leading causes of myocarditis. Immune-suppression after chemotherapy increases opportunistic infections, but the incidence of virus-induced myocarditis is unknown.

**Objective:**

An unbiased, blinded screening for RNA viruses was performed after chemotherapy with correlation to cardiac function.

**Methods:**

High-throughput sequencing of RNA isolated from blood samples was analyzed following chemotherapy for hematological malignancies (*N* = 28) and compared with left ventricular ejection fraction (LVEF).

**Results:**

On initial rigorous analysis, low levels of influenza orthomyxovirus and avian paramyxovirus sequences were detectable, but without significant correlation to LVEF (r = 0.208). A secondary broad data mining analysis for virus sequences, without filtering human sequences, detected significant correlations for paramyxovirus with LVEF after chemotherapy (r = 0.592, *P* < 0.0096). Correlations were similar for LVEF pre- and post- chemotherapy for orthomyxovirus (R = 0.483, *P* < 0.0421). Retrovirus detection also correlated with LVEF post (r = 0.453, *p* < 0.0591), but not pre-chemotherapy, but is suspect due to potential host contamination. Detectable phage and anellovirus had no correlation. Combined sequence reads (all viruses) demonstrated significant correlation (r = 0.621, *P* < 0.0078). Reduced LVEF was not associated with chemotherapy (P = NS).

**Conclusions:**

This is the first report of RNA virus screening in circulating blood and association with changes in cardiac function among patients post chemotherapy, using unbiased, blinded, high-throughput sequencing. Influenza orthomyxovirus, avian paramyxovirus and retrovirus sequences were detectable in patients with reduced LVEF. Further analysis for RNA virus infections in patients with cardiomyopathy after chemotherapy is warranted.

## Introduction

The use of chemotherapy in treatment for neoplastic disease is associated with frequent side effects and non-therapeutic toxicities (1–3). Chemotherapy associated cardiac toxicity is well-described for some chemotherapeutic agents, however, for many agents the cause of cardiac toxicity is not defined (1–3). Chemotherapy-associated heart failure has considerable associated mortality and morbidity. Amongst 3,234,256 cancer patients in the United States, 38% have died from cancer and about 1 in 3 have died from cardiovascular disease (CVD). Among the deaths from CVD, 76% were in patients younger than 35 years. The incidence of all chemotherapy-associated cardiotoxicities is reported to be ~10% in all treated patients, and this is projected to increase as more patients receive chemotherapy (1–3).

Chemotherapy induces leukocyte cytotoxicity and immune suppression with increased risk of opportunistic infections, including bacteria, fungi and viruses (4–6). Viruses are the most common cause for myocarditis in patients without cancer or chemotherapy (7–9) and myocarditis is linked to a wide array of viruses, in some cases with severe heart failure (10–17). While opportunistic bacterial and fungal infections are frequently reported after chemotherapy and immunosuppression, opportunistic viral infections are less often reported. Herpesvirus, hepatitis B/C, influenza and parainfluenza viruses, respiratory syncytial virus (RSV), and retrovirus infections have been reported after chemotherapy (7–9), but the role of viruses in myocarditis in immunosuppressed patients after chemotherapy has not been studied. Opportunistic viral infection after chemotherapy is thus predicted to contribute to heart damage and heart failure.

Among the RNA viruses, Coxsackie virus B3 (CVB3), human parvovirus B19 (B19V), measles, retroviruses and influenza viruses have been implicated in myocarditis among other viruses, and these viruses have been detected after chemotherapy (7–16). B19V and the enterovirus, coxsackievirus, are considered leading causes of viral myocarditis (7–15). DNA viruses are also linked to myocarditis, specifically cytomegalovirus (CMV), Epstein bar virus (EBV), human herpes simplex virus-6 (HHV6) also cause myocarditis (17–19). In the past year, the RNA virus, Severe Acute Respiratory Syndrome Coronavirus 2 (SARS-CoV-2) infection has also been implicated in acute myocarditis (14–16). Viral infections thus present a risk for myocarditis after immune suppression with chemotherapy.

In patients with chronic unexplained heart failure positive for B19V and treated with intravenous immunoglobulins (IVIG), a significant decrease in B19V load has been observed with improved cardiac function, symptoms, and decreased end diastolic volume (8). Other treatments that selectively target specific viruses, such as acyclovir that inactivates herpes simplex virus (HSV) DNA polymerase for herpes myocarditis or antiretroviral therapy for human immunodeficiency virus (HIV) infections have proven beneficial (8–19). Development of new vaccines to target viral infections are highly effective as for SARS-CoV-2 and COVID-19, but viruses with the potential to cause chemotherapy induced myocarditis must be identified in order to develop effective vaccines and treatments.

Accordingly, we postulated that immune suppression caused by chemotherapy, or by the cancer itself, will increase susceptibility to viral infections, and therefore myocarditis. This study presents an unbiased, blinded screen for RNA viruses in blood samples obtained from patients after chemotherapy for hematological malignancies. In this pilot study, reductions in measured left ventricular ejection fraction (LVEF) on 2D echocardiogram were correlated with measured levels of detected RNA virus gene signatures. To the best of our knowledge, this is the first screen for detectable RNA virus sequences in blood samples from cancer patients with evidence for myocarditis and reduced LVEF after chemotherapy. Information on opportunistic viral infections seen after immune suppression and chemotherapy will foster a defined preventative approach, such as development of vaccines or treatment with specific anti-viral agents designed to reduce the risk of cardiac damage.

## Materials and Methods

### Patient Population

Twenty-eight patients were enrolled in the Cardio-Oncology (AL) and the Hematologic Malignancies / Stem Cell Transplantation clinics (SC, JM) at the University of Florida after written informed consent. All patients and samples were assigned a randomized number after informed consent. The study was approved by the Institutional Review Boards (IRB) at all institutions involved in the study.

**Figure 1:**
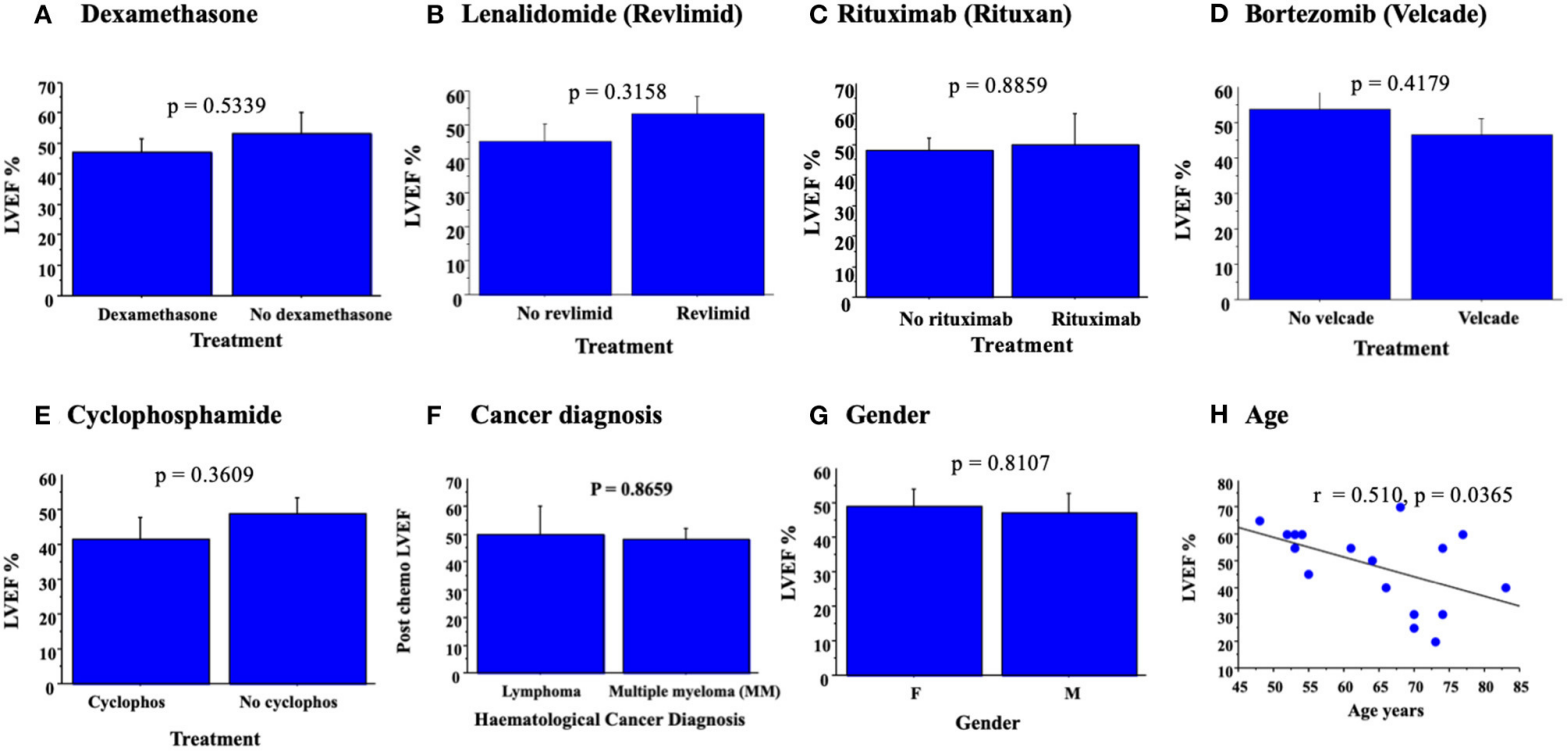
Bar graphs demonstrating mean LVEF ± SE with chemotherapy. No significant change in LVEF, as assessed by ANOVA, was detected for treatments with dexamethasone **(A)**, revlimid **(B)**, lenolidomide **(C)**, bortezomib **(D)**, cyclophosphamide **(E)**, cancer diagnosed **(F)**, or gender **(G)**. Simple regression analysis demonstrates a significant correlation between LVEF post chemotherapy and increased age **(H)**.

Patient ages ranged from 18 to 85 years; all had a diagnosis of cancer and a range of chemotherapeutic agents including dexamethasone, lanolidomide, rituximab, bortezomib, and cyclophosphamide, as well as combinations as noted (Table 1; Figure 1). The majority of patients had a history of hematological malignancy (25/26 hematological malignancy and 1/26 breast cancer) (Table 1). Six patients had a history of prior diagnosed ischemic heart disease, one with documented coronary artery disease and stent implant. Of the enrolled patients, 26 had complete viral sequencing (ST, SS-C) (Table 1). Eighteen patients had LVEF measured post chemotherapy, 22 patients had LVEF measured before chemotherapy, and 16 had both. One patient had confirmation of LVEF by cardiac catheterization. Chemotherapy given was recorded for each patient. Echocardiograms were ordered by the attending physician based upon perceived clinical risk, but were not mandated. Cardiac dysfunction was defined as a decrease in ejection fraction (EF) <45% as demonstrated on echocardiography. LVEF data was available but more comprehensive data such as fractional shortening was not provided.

**Table 1:** Study patient demographics.

**Patient ID**	**Diagnosis**	**Age**	**Chemotherapy**	**Repeat chemotherapy**	**Stage**	**Diagnosis of prior heart disease**	**Ejection fraction (EF) prior to chemotherapy**	**Ejection fraction (EF) post chemotherapy**
PJ0266_R2	Multiple myeloma (MM)	70–85	Velcade and dexamethasone (2010–2012) –> 12/7/2012 (ASCT)–>revlimid maintainance (2013 till 1/2015)	None	IIIA	No	60	30
PJ0267_R2	Follicular lymphoma	80–85	Bendamustine and rituximab(1/19/2016–4/12/2016) f/b rituximab 8/2016–1/2018)	Rituximab	IIA	IHD	60	40
PJ0268_R2	Multiple myeloma (MM)	60–65	Revlimid, velcade, dexa (1st)–> CVD	CVD,	IIIB	No	60	50
PJ0269_R2	Chronic lymphocytic leukemia (CLL)	75–80	Ibrutinib	NA	NA	IHD	60	NA
PJ0270_R2	Multiple myeloma (MM)	70–75	Revlimid, velcade	NA	IIIA	IHD	60	NA
PJ0271_R2	Multiple myeloma (MM)	70–75	Velcade, dexamethasone–> pomalidomide, dex	NA	IA	No	60	NA
PJ0272_R2	Mantle cell lymphoma	50–55	Rituximab, fludarabine, cyclophosphamide-> busulfan, cyclophosphamide, vincristine (conditioning)_-> rituximab maintenance	NA	IVA	No	60	60
PJ0273_R2	Multiple myeloma (MM)	50–55	Velcade, revlimid, dexa (2014)–> revlimid (2015, 2016)–> melphalan and transplant	Revlimid, transplant	NA	No	NA	60
PJ0274_R2	Multiple myeloma (MM)-kappa LC	45–50	RVD	RVD, ASCT, velcade	IIIA (II)	No	60	65
PJ0275_R2	Multiple myeloma (MM)-IgG kappa	70–75	CTX/velcade	Dexa, CTX, VCR, Mel,	IIA (I)	IHD	30	40
PJ0276_R2	Multiple myeloma (MM)-IgG lambda	70–72	RT, CVD	Dara/Velcade/Dexa	IIIA	No	60	NA
PJ0277_R2	Multiple myeloma (MM)-IgG kappa	50–55	RVD-CVD, revlimid maitenance, relapse #1, Kyprolis, pomalyst, VBCP	VBCP, Dara	IA (I)	No	55	55
PJ0278_R2	Multiple myeloma (MM)-KLC	50–55	CVD	Revlimid, velcade, melflufen, Dexa	IIIB (III)	No	35	45
PJ0279_R2	Multiple myeloma (MM)-PCL-LLC	65–70	CVDX 4	Revlimid, prednisone	NA	No	60	40
PJ0280_R2	Multiple myeloma (MM)-KLC	65–70	CVD X 4	Revlimid	IIIA (I)	No	60	ND
PJ0281_R2	Multiple myeloma (MM)-KLC	65–70	VD X 6	None	IB (II)	No	NA	70
PJ0282_R2	Multiple myeloma (MM)-IgG kappa	75–80	VTD, RVD	CVD, PD, PD+Elo, Dara, Dara+Velcade	IIIA (?)	No	60	60
PJ0283_R2	Multiple myeloma (MM)-IgA Lambda	65–70	CVD	Revlimid maintenance	IIIA (II)	No	60	ND
PJ0284_R2	Multiple myeloma (MM)-IgG lambda, Acute lymphocytic leukemia (ALL)	60–65	VD	Revlimid maintenance	IIIA (II)	No	60	55
PJ0285_R2	Multiple myeloma (MM)-IgG kappa	70–75	CVD	Revlimid, RVD, Kyprolis, pomalidomide	IIIA (?)	No	55	20
PJ0286_R2	Multiple myeloma (MM)-IgG lambda	70–75	Thal/Dexa	Revlimid, CTX, RCD, high dose kyprolis	IIIA (II)	No	60	55
PJ0287_R2	Multiple myeloma (MM)-IgG kappa	70–75	VAD	Revlimid maintenance, velcade/pomalidomide/dexa Dara, CTX, CVD,PD, VBCP,Kyprolis, VTD-PACE		No	55	25%
PJ0288_R2	Cirrhosis, DCIS, CAD	50–55	TAM	NA		IHD	65	60
PJ0289_R2	Multiple myeloma	70–75	Velcade and dexa (2010–2012) –> 12/7/2012 (ASCT)–>revlimid maintainance (2013 till 1/2015)	NA		No	NA	NA
PJ0290_R2	Follicular lymphoma	80–85	Bendamustine and rituximab(1/19/2016 - 4/12/2016) f/b rituximab 8/2016- 1/2018)	Rituximab	IIA	IHD	60%	40–45%
PJ0291_R2	Multiple myeloma	60–65	Revlimid, velcade, dexa (1st)–> CVD	NA		NA	NA	NA

*dexa, dexamethasone; RVD, revlimid, velcade, dexa; ASCT, autologous stem cell transplant; CTX, cyclophosphamide; DCIS, ductal carcinoma in situ; VCR, vincristine; CVD, velcade, cyclophosphamide, dexa; Mel, melphalan; Dara, daratumumab; VD, velcade and dexamethasone; RT, radiation therapy; VBCP, vincristine, BCNU, cyclophosphamide, and prednisone; Thal/Dex, thalidomide and dexamethasone; PD, pomalidomide and dexamethasone; PD+ Elo, PD and Elotuzumab; VTD, velcade, thalidomide and dexa; VAD, vincristine, Adriamycin and dexamethasone; RCD, revlimid, cyclophosphamide and dexa; VTD-PACE, Velcade, thalidomide, dexamethasone, platinum, Adriamycin, cyclophosphamide, and etoposide; TAM, tamoxifen*.

### Virome Sequencing of Blood Samples

Blood samples were obtained 2–4 weeks after chemotherapy, once informed consent was obtained. Venous blood was collected in clinic at 2–4 weeks after the last chemotherapy in tubes containing 3 mL of Ethylenediaminetetraacetic acid (EDTA). RNALater (Life Technologies, Bleiswijk, The Netherlands) was added at a ratio of 2:1 RNALater to whole blood and samples stored at −80°C until analysis. Blood samples were anonymized blood samples and were sent to St Jude's Hospital (SCC, ST); where investigators were masked to cancer type, chemotherapy, and LVEF findings.

Samples were fragmented prior to RNA extraction as previously described (20). RNA was isolated using the QIAamp cador Pathogen Mini kit (Qiagen, Hilden, Germany) according to manufacturer's instructions. First strand synthesis was completed using the random-primer technique described by Wang et al. (21). Briefly, Super Script III (Invitrogen, Carlsbad, CA, USA) first strand synthesis was carried out using primer A (5'-GTTTCCCAGTCACGATANNNNNNNNN), followed by Sequenase (Affymetrix, Santa Clara, CA, USA) for second strand synthesis. Finally, PCR amplification utilized Primer B (5'-GTTTCCCAGTCACGATA) for 40 cycles. Samples were electrophoresed in an agarose gel to confirm product.

Samples were run on a 1% agarose gel and imaged to confirm the presence of DNA (between 500 bp and 1 kb). To prepare for sequencing, samples were purified using AMPure XP beads (Beckman Coulter, Brea, CA, USA) followed by analysis on a Qubit fluorometer (Thermo Fisher Scientific, Waltham, MA, USA). A Nextera DNA library preparation kit (Illumina, San Diego, CA, USA) was used for sample genome sequencing using the Illumina MiSeq Amplicon application for virome reference genomes. Quality control of input nucleic acid and final libraries were checked using Agilent TapeStation 4200.

### Bioinformatics Analysis

In an initial analysis, Illumina sequencing data sets were examined for viral pathogen detection. The quality of raw reads was assessed using FastQC (version 0.11.9) (https://www.bioinformatics.babraham.ac.uk/projects/fastqc/). Trimmomatic was used to trim the adapters from the paired-end reads (22). Taxonomic classification was performed for blood samples using Kraken2 (version 2.0.7b). Illumina sequencing data sets were initially analyzed for detection of RNA viruses (23–25). The RNA viral reads that were viral-like were also checked using BLASTn against a viral RefSeq database. Sequencing data is available, Bioproject accession number NCBI, PRJNA794842, temporary submission ID SUB10902989, release date: 2022-01-06.

For the broader viral RNA sequence analysis (SS-C, ST), a custom viral database, containing complete viral genomes cross referenced to RefSeq (ftp://ftp.ncbi.nlm.nih.gov/refseq/release/) with taxonomic classifications, was built using Kraken2 (26). Following that, Kraken reports were analyzed, visualized and summarized using Pavian [(27), Supplementary Figure 1]. The database used for taxonomic classification included all viral genomes in RefSeq. Contigs sequences were used to identify the viral genetic sequences. To verify that human endogenous retrovirus K (HERV K) is detected correctly, we extracted the raw reads that classify as HERV K. The extracted raw retrovirus K reads were then mapped to human endogenous retrovirus reference genome (NC_022518.1) and human reference genome (GRCh38) separately. The quality of mapping to both reference genomes was assessed.

### Statistical Analysis—Correlation of Viral Genetic Sequences With Cardiac Function and Chemotherapy

Levels of viral gene sequences detected in blood samples were correlated with heart function (LVEF) before and after chemotherapy using simple regression analysis with confirmation by our statisticians (PM,AV,SK at ASU; ST, SS-C at St Judes). All statistics were performed using Statview, version 5.01 (SAS, Inc., Cary, NC) or Graphpad Prism (GraphPad). Multiple-group comparisons were performed using analysis of variance (ANOVA) with Fishers protected least significant difference (PLSD) and unpaired two-tailed Student's *t-*test for subgroup analysis. A *P* < 0.05 is considered significant.

## Results

### Analysis of Associations Between Cardiac Function and Chemotherapy

No significant correlation was detected for the specific chemotherapy given to each patient and LVEF post chemotherapy, measured as LVEF (Table 1); *p* = 0.5339 for dexamethasone (Figure 1A), *p* = 0.3158; lenolidamide (Figure 1B), *p* = 0.3158; rituximab (Figure 1C), *p* = 0.8859; bortezomib (Figure 1D), *p* = 0.4179; and cyclophosphamide (Figure 1E), *p* = 0.3609. When a chemotherapeutic agent was used in only one of the patients, this was considered an inadequate number to allow analysis for correlation with LVEF. Analysis of changes in LVEF with specific cancer diagnosis demonstrated no significant correlation between diagnosed cancer type and LVEF (Figure 1F, *p* = 0.8659). No significant association was made between patient sex and measured LVEF (*p* = 0.8107, Figure 1G). Simple regression analysis did demonstrate a significant correlation between an increased age of patients and reduced LVEF, both pre and post chemotherapy (for post chemotherapy—r = 0.510, *p* < 0.0365) (Figure 1H). The minority of patients were receiving cardiovascular medications at enrollment, 6/ 26 patients had diagnosis of IHD listed (Table 1; ANOVA *P* = 0.056) and diagnosed IHD and CVD medications were not associated with changes in LVEF (*p* = 0.8659, Figure 1H).

### High Throughput Sequencing of RNA Virus in Blood Samples From Patients

Low levels of RNA virus sequences were detected in blood samples isolated from patients post chemotherapy. Detection of virus sequences using a strict, blinded analysis with either Blastn or Kraken 2 programs, and removing all potential contaminating human sequences, detected low levels of paramyxovirus and orthomyxovirus, as representative of potential opportunistic infections. The avian Avulavirus, a paramyxovirus, and Influenza A, an orthomyxovirus, were consistently identified on sequence analysis using both methods for RNA Seq analysis (Blastn and Kraken 2), indicating low levels of these RNA viruses after chemotherapy. The paramyxovirus avian Avulavirus and the orthomyxovirus Influenza A were the most frequently identified sequences.

### Effects of Chemotherapy on Strict RNA Virus Sequence Read Detection

**Figure 2:**
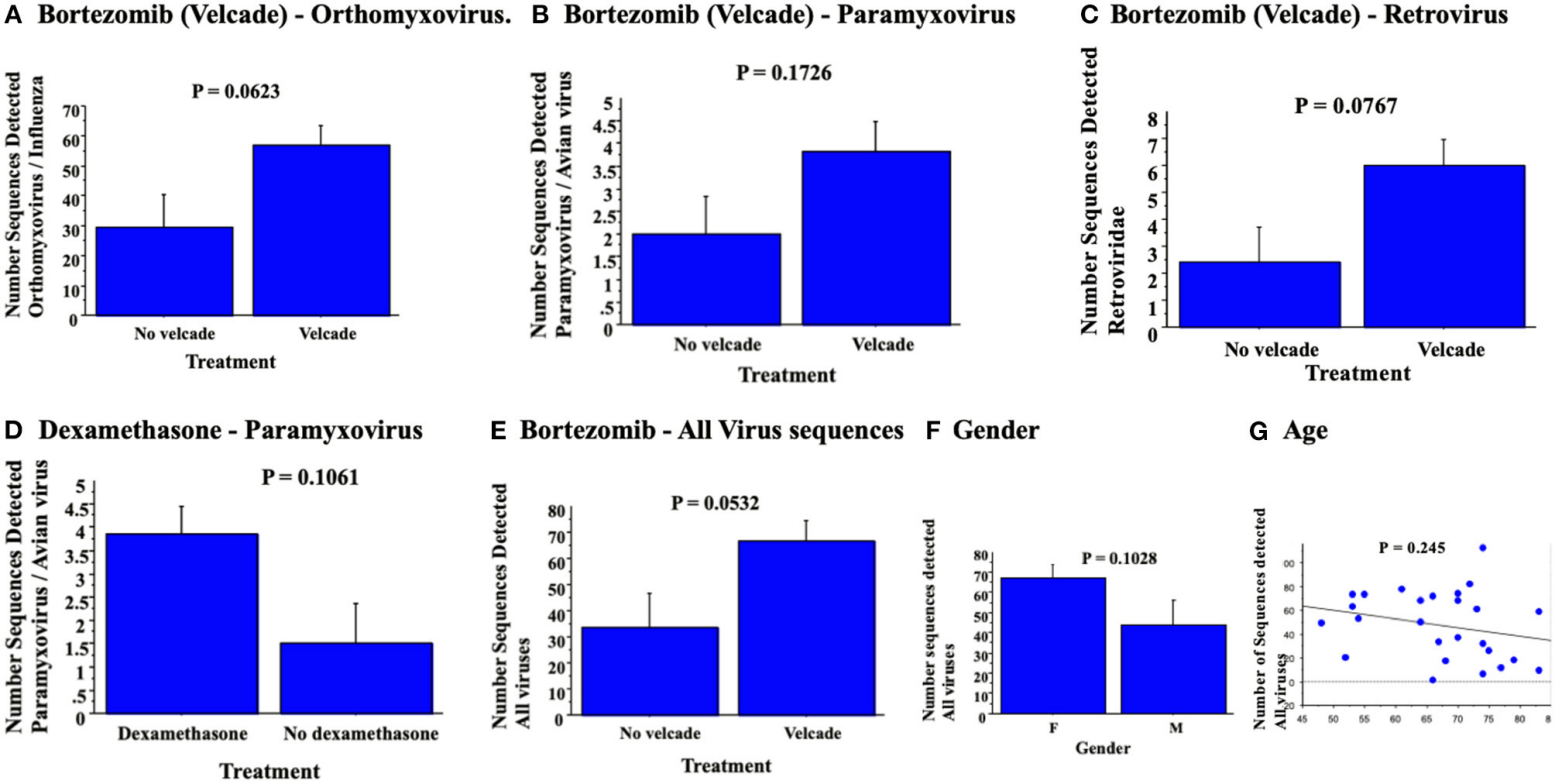
RNA virus sequence reads detected in blood samples demonstrated no significant association for orthomyxovirus **(A)**, paramyxovius **(B)** nor retrovirus **(C)** with bortezomib or with dexamethasone for detected paramyxovirus reads **(D)**. Bortezomib chemotherapy was associated with a trend toward detection of all paramyxo-, orthomyxo- and retro-virus sequences detected **(E)**. Analysis for virus detection with gender (**F**, ANOVA) and age (**G**, simple regression analysis) detected no significant changes.

Potential correlations were assessed for detected RNA virus sequences and treatments with individual chemotherapeutic agents. Levels of virus sequences detected were increased with bortezomib and dexamethasone (Figure 2), however, there was only a trend toward increased detection of virus sequences in blood samples; *p* = 0.0623 for orthomyxovirus detection (Figure 2A), *p* = 0.1726 for paramyxovirus detection (Figure 2B) and *p* = 0.0767 for retrovirus sequences (Figure 2C) after bortezomib and *p* = 0.1061 for paramyxovirus (Figure 2D) after dexamethasone, none reaching significance. Analysis of a combined count of all detected RNA virus sequences in patients with bortezomib chemotherapy again detected a borderline increase (*p* = 0.053) (Figure 2E). Lenolidomide and cyclophosphamide treatment had no clear trend nor significant change in RNA virus gene sequence detection. In contrast, rituximab treatment was associated with a non-significant trend toward decreased virus sequence detection (not shown). Detection of RNA virus sequences in blood samples from cancer patients was not linked to gender (*p* = 0.1028, Figure 2F) nor age (*p* = 0.245, Figure 2G).

### Initial Strict Analysis of RNA Virus Sequence Detection and Cardiac Function After Chemotherapy

**Figure 3:**
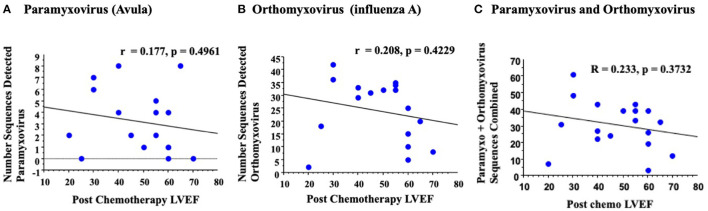
An initial rigorous analysis for detected virus sequences in blood samples with reduced LVEF detected no significant correlation. Analysis of paramyxovirus **(A)** orthomyxovirus **(B)** or total combined paramyxovirus and orthomyxovirus **(C)** sequences detected no significant correlation on simple regression analysis.

On an initial rigorous, or strict, analysis, removing all potential contaminating human, bacterial or phage sequences, RNA virus sequences in the paramyxovirus (28) and orthomyxovirus (29) families were detected. Virus sequence detection was read blinded by four investigators (ST, SS-C, AV, AL). No significant correlations were detected for measured LVEF post chemotherapy; r = 0.177 for paramyxovirus (*p* = 0.4961, Figure 3A), and r = 0.208 for orthomyxovirus (*p* = 0.4229, Figure 3B). Screening for LVEF and a combined analysis of paramyxovirus and orthomyxovirus virus sequences detected a minimal, but again non-significant, increase in the correlation with reductions in LVEF reported on 2D echo post chemotherapy (r = 0.233, *p* = 0.3732, Figure 3C). This would suggest that RNA virus gene signatures are detectable at low levels in blood samples from patients with reduced LVEF post chemotherapy, but do not have a significant correlation for reduced LVEF for individual detectable viruses in this small patient cohort.

### Secondary Custom Database Analysis of RNA Virus Sequence Detection and Cardiac Function After Chemotherapy

A secondary, broad, customized Kraken2 database was next designed to detect a wider spectrum of RNA virus sequences as a metagenome analysis platform. This secondary analysis was performed without rigorous filtering of potential human genome sequences, and accepting both unidirectional as well as bidirectional reads. With this broad analysis an increased correlation was detectable for RNA virus sequences (Illustrated via Pavian software, Supplementary Figure 1). Bidirectional reads were used for analysis of correlations between detected virus gene signatures with measured LVEF post chemotherapy. Correlations of virus sequence detection with LVEF recorded prior to chemotherapy was used as a control to assess changes in LVEF prior to treatment with chemotherapy.

**Figure 4:**
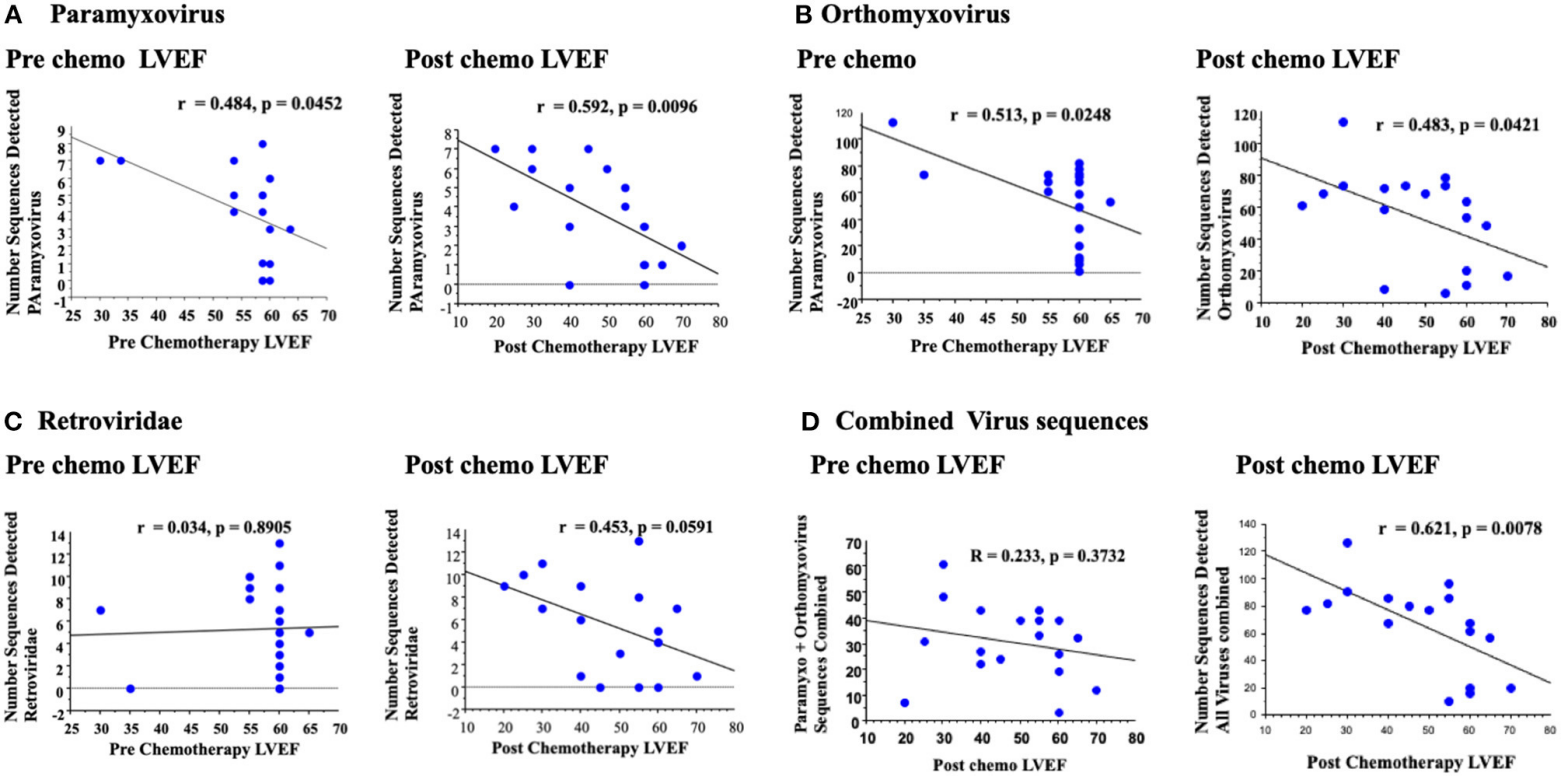
A secondary broad analysis demonstrated a correlation between paramyxovirus bidirectional sequence reads with LVEF pre and post chemotherapy, but with greater correlation and significance post chemotherapy LVEF **(A)**. Similar correlations were detected for orthomyxovirus both pre and post chemotherapy **(B)**. Retrovirus reads had no detectable correlation with LVEF pre chemotherapy, but a trend to increased correlation post chemotherapy **(C)**. Analysis of all RNA virus reads for paramyxovirus, orthomyxovirus and retrovirus reads detected a significant correlation for changes in LVEF post chemotherapy **(D)**.

Paramyxovirus sequence detection and retrovirus sequence detection, using bidirectional reads from this more permissive analysis, had increased correlation when comparing LVEF post chemotherapy and LVEF measured pre chemotherapy; For paramyxovirus the correlation increased from r = 0.484 pre chemotherapy LVEF (*P* < 0.0452) to r = 0.592 for LVEF measured post chemotherapy (*P* < 0.0096, Figure 4A), with a clear increase in significance. Albeit both achieve significance.

Conversely, orthomyxovirus sequence detection, specifically for influenza A, had similar correlations when comparing paramyxovirus sequence reads to LVEF measured prior to or after chemotherapy (r = 0.513 pre, *P* < 0.0248 and r = 0.483 post, *P* < 0.0421). Although both analyses for influenza A reached significance prior to or after chemotherapy, the fact that there was a significant and greater correlation prior to chemotherapy would suggest no specific association between orthomyxovirus detection and developing cardiac dysfunction after chemotherapy. Overall, orthomyxovirus and paramyxovirus sequences were again detected in this broad, less strict analysis, but with increased numbers of detectable reads using this custom platform.

**Figure 5:**
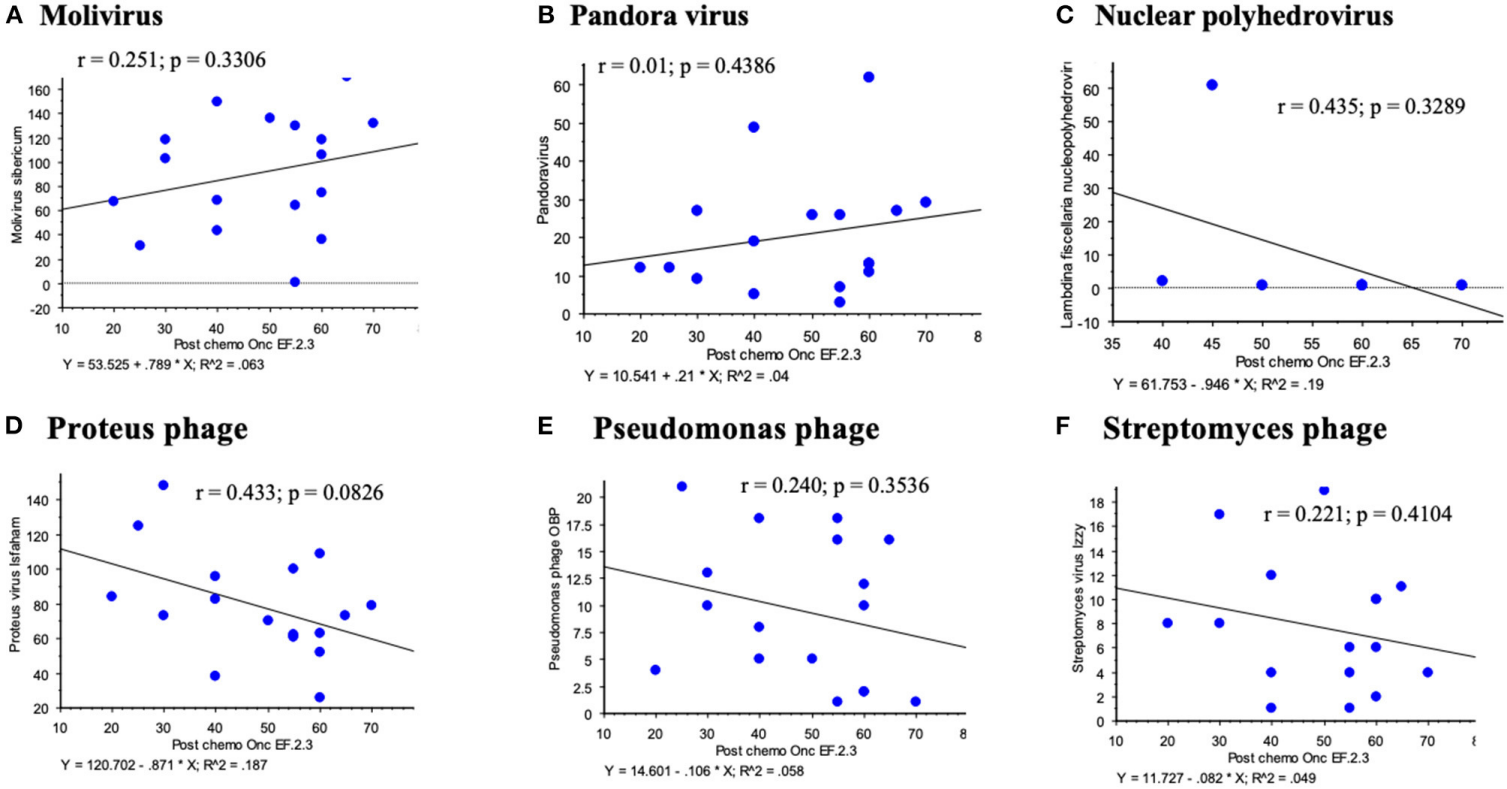
Other virus sequences detected on the broader platform for RNA viruses demonstrated no significant correlations between viral sequence reads and LVEF post chemotherapy; Molivirus **(A)**, Pandora virus **(B)**, Panderina fiscellaria nucleopolyhedrovirus **(C)**, Proteus **(D)**, Pseudomonas phage **(E)** and Streptomyces **(F)** phage.

Other RNA virus sequence reads were also detected using this broad analysis (Figure 5). One of the most prominent was the retrovirus HERV K (30). For retrovirus sequences, the correlation increased from r = 0.034 for LVEF measured pre chemotherapy (*P* = 0.8904, non-significant) to r = 0.453 post chemotherapy with a borderline trend toward significance (*P* = 0.0591, Figure 5C). Retrovirus sequences detected were predominantly human endogenous retrovirus K (HERV K), a retrovirus commonly detected in the human genome. Retrovirus sequences may represent activation from the native human cell genome, during the stress of cancer, chemotherapy or extrinsic infections.

Among other sequences detected there were Molivirus, Pandoravirus and Pandoravirus dulcis, Lambdina fiscellaria nucleopolyhedrovirus, Leukania separata nucleopolyhedrovirus, and several phage including Proteus virus mirabilis Isfaham, Aeromonas virus 25AhydR2PP and Aes 12, Caulobacter virus CcrBL10, Vibrio phage Eugene 12A 10, Streptomyces virus Izzy, and Pseudomonas phage OBP and PAJU2. None of these additional virus sequence reads demonstrated significant correlations for changes in LVEF post or even pre chemotherapy for hematological cancers (Figure 5 illustrates some of these analyses). For these virus sequences compared to LVEF post chemotherapy, the following r values were obtained: Molivirus r = 0.251, *P* = 0.3306; Pandoravirus and Pandoravirus dulcis r = 0.015, *P* = 0.9538; Lambdina fiscellaria nucleopolyhedrovirus r = 0.435, *P* = 0.3289; Leukania separata nucleopolyhedrovirus r = 0.114–0.201, *P* = 0.4372–0.6725; and several phage including Lactococcus phage BK5-T r = 0.228, *P* = 0.3625; Streptomyces virus Izzy r = 0.221, *P* = 0.4104; Proteus mirabilis virus Isfaham r = 0.433, *P* = 0.0826; Aeromonas virus 25AhydR2PP r = 0.447, *P* = 0.1088; Aes 12 r= 0.124, *P* = −0.6483; Caulobacter virus CcrBL10 r = 0225, *P* = 0.3849; Vibrio phage Eugene 12A 10 r = 0.376, *P* = 0.5429, Pseudomonas phage OBP and PAJU2, r = 0.240–0.356, *P* = 0.1927–0.3536. A circular DNA Anellovirus, signature was detected in one patient with multiple myeloma and demonstrated no correlation.

A potential correlation between changes in LVEF pre and post chemotherapy with an analysis of a combined RNA virus sequence reads for virus sequence detection where trends toward increased RNA viral sequence correlations with reduced LVEF were demonstrated was then examined. A combined read for detcted paramyxo- orthomyxo- and retro-virus sequnces was assessed using this broad screen (Figure 4D). On this final analysis of combined RNA virus sequences for each sample, the correlation increased from r = 0.233 (*p* = 0.3732) for pre chemo LVEF to r = 0.621 (*P* < 0.0078) for post chemo LVEF, a markedly significant increase. Thus, increased detection of three detectable RNA virus sequence reads, paramyxo, orthomyxo- and retro-viruses after chemotherapy did demonstrate an apparent significant association with measured reductions in LVEF after chemotherapy (Figure 4D).

## Discussion

Dilated cardiomyopathy in cancer patients is a critically important complication after chemotherapy produced by cardiotoxicity with associated increased mortality (1–3). Immunosuppression with secondary opportunistic viral infection increases the risk for virus-induced myocarditis and heart damage after chemotherapy (4–9). Individual chemotherapeutic regimes have proven cardiotoxicity, but there are multiple molecular mechanisms for cardiac damage that differ for individual chemotherapeutic agents and some have no known etiology for cardiotoxicity (31–35). Viral infections are a leading cause for myocarditis in patients without cancer or chemotherapy (7–16, 36) and immunosuppression after chemotherapy increases the risk for opportunistic viral infections. We have performed a pilot study to screen for potential correlations between RNA virus sequences detected in blood samples and reduced LVEF post chemotherapy in patients with hematological tumors.

Chemotherapy is used for both hematological cancers and solid tumors with proven cytotoxicity, with a variety of non-therapeutic and potentially adverse side effects. Myocardial toxicity leads to left ventricular dysfunction (LVD), heart failure (HF), endothelial dysfunction, thrombogenesis, ischemia, vasospasm, pericardial disease, hypertension, and rhythm disturbances (1–3, 31–35). Incidence and cardiotoxic effects vary with individual chemotherapy agents. The incidence of LVD is 3–26% for Doxyrubicin (anthracycline), 7–28% for Cyclophosphamide, 17% for Ifosfamide, 1–3% for Bevacuzimab 2–28% for Trastuzumab therapy and 0.5–1.7% for Imatinib mesylate therapy (1, 2). Causes for cardiac toxicity varies from direct cardiomyocyte toxicity to vascular injury. Several mechanisms have been proposed for each class of agent, but individual chemotherapeutic reagents have differing molecular targets and reported causes for heart damage; there is, as might be expected, no unifying mechanism. The mechanism underlying anthracycline-induced cardiotoxicity has been reported as interference with topoisomerase II beta and secondary free-radical formation; apoptosis; transcriptional changes in intracellular adenosine triphosphate (ATP); reduced sarcoplasmic reticulum calcium-ATPase expression; prolonged depression in cardiac glutathione peroxidase activity; and respiratory defects associated with mitochondrial deoxyribonucleic acid damage (12, 31–35). Cyclophosphamide-induced cardiotoxicity is postulated to be caused by endothelial injury through toxic metabolites that damage cardiomyocytes and secondary intracapillary microemboli and coronary vasospasm (1, 2). The mechanism of HF associated with bevacizumab is believed to be associated with uncontrolled HTN and inhibition of vascular endothelial growth factor (VEGF)/VEGF receptor signaling (1–3). Damage to the myocardium after treatment with chemotherapy thus has differing etiologies dependent upon the underlying cancer, treatment used, and the cause is incompletely defined for many newer chemotherapies. The potential for viral infection and secondary myocarditis after immunosuppression with chemotherapy for cancer has not been systematically examined as a potential cause for cardiomyopathy after chemotherapy.

In this study we measured RNA virus sequences in blood samples obtained from patients after chemotherapy using RNA Seq screening, RNA isolates were assessed both by an initial rigorous analysis that removed any potential human sequence contamination as well as with a subsequent broader, customized virome analysis. This was an unbiased screen for the detection of RNA virus sequences in venous blood samples in cancer patients receiving chemotherapy. On the initial more rigorous analysis, both paramyxovirus and orthomyxovirus sequences were detectable, but had no clear correlation with changes in LVEF (Figure 3). The orthomyxovius Influenza A has a well-known association with viral myocarditis, but demonstrated similar correlations with LVEF measured prior to, or after, chemotherapy (6–9). Both paramyxovirus and orthomyxovirus families have been reported as causes for myocarditis in patients without known prior cancer or chemotherapy. The detectable sequences for orthomyxovirus matched to influenza A (29) and those for paramyxovirus with avulavirus (28). On a secondary broad analysis using a customized screen, we were able to detect larger numbers of RNA virus sequences for orthomyxovirus influenza A and paramyxovirus Avulavirus, as well as a larger array of RNA viruses including retroviruses, insect viruses and phage. In this second analysis we identified correlations between the detected paramyxovirus and retrovirus with changes in LVEF.

The increase in the paramyxovirus Avulavirus, sequences does demonstrate an increase in sequence detection and reduced LVEF after chemotherapy, with a greater correlation for post chemotherapy LVEF than for pre-chemotherapy LVEF. Orthomyxovirus influenza A was also detected, but with similar detected sequence counts and correlations for LVEF measured pre- or post-chemotherapy (Figure 4B); this correlation was stronger for pre-chemotherapy. Influenza is a common upper respiratory infection and the detection of this orthomyxovirus may be attributed to the prevalence of influenza A virus in the general population, with a generalized increase in an immunocompromised cohort. This suggests a general association of influenza A, the orthomyxovirus, with a reduction in LVEF that is unrelated to chemotherapy. The paramyxovirus Avulavirus sequence detection was also detectable, but the correlation was greater for LVEF measured after chemotherapy, albeit a small increase (Figure 4A). On the secondary, broader analysis, the greatest increase in correlation was seen with the HERV K retrovirus sequence on the post chemotherapy sample. Simple regression analysis for pre-chemotherapy LVEF analysis exhibited a flat, unresponsive regression with no evidence for correlation with LVEF pre chemotherapy, but with a clear inverted, negative correlation for detected HERV K sequences with post-chemotherapy LVEF (Figure 4C).

Retroviruses are reported to represent up to 8% of the human genome (37–41). Retrovirus sequence detection may represent activation of latent human retroviruses rather than opportunistic infection, as has been reported. Retrovirus K reads may represent reactivation from the human genome induced by the stress from chemotherapy and/ or cancer. Blast confirmation indicated that all reads belonged to the human genome suggesting reactivation rather than *de novo* infections. HERV-K is the most transcriptionally active retrovirus in man, representing up to 8% of the human genome, and thus may also represent a contamination. HERV-K has been associated with neurodegenerative disorders, cancer, and an overall higher tumor burden (38–41). HERV K genes, like nuclear protein-1, has important roles in the generation of reactive oxygen species and other tumorigenic characteristics that may act in synergy with chemotherapeutics to decrease LVEF. HERV-K has also been reported to be upregulated after chemotherapy and could suggest that the stress of chemotherapy or the immunosuppression may have induced increased HERV-K expression.

Phage infect bacteria and alter bacterial responses after chemotherapy. We detected some phage sequences and these might be considered contaminants, as are the pandoraviruses. However, recent reports have demonstrated high levels of phage in many organs outside the gut (42, 43). Increased levels of phage are reported in the circulating blood in patients with increased gut permeability in leukemic patients where “leaky gut” is suspected to allow phages to translocate from the gut to the plasma (43). In this study, no correlation was uncovered between the detected phage sequences and insect virus reads with LVEF post chemotherapy (Figure 5). No correlation was found between anellovirus and reduced LVEF. These identified virus reads likely represent incidental detection of DNA viruses during analysis of RNA virus cDNA.

Bortezomib was associated with a borderline increase in detection of viral sequences (*P* = 0.0532). Other than age, none of the other parameters, chemotherapeutic agent, gender, cancer diagnosis and none of the other chemotherapeutic drugs demonstrated clear correlations with reduced LVEF pre- or post-chemotherapy.

This study was realized as an unbiased survey, a pilot study, to detect RNA virus sequences in blood samples in post chemotherapy patients with hematological cancers. In this study we were able to detect paramyxovirus, orthomyxovirus, and retroviruses in venous blood samples taken post chemotherapy for hematological malignancy. With a broad analysis, correlations were detected with LVEF measured post chemotherapy. This is, however, limited to a correlation without a definitive cause-and effect. A comprehensive analysis of correlations between detected virus sequences and reduced LVEF with scheduled blood samples, LVEF analysis, and heart damage markers prior to and after chemotherapy should be considered and has the potential to demonstrate chemotherapy induced immune suppression and the risk of heart damage due to viral infection and/or reactivation.

## Limitations of Study

This study is limited by the small numbers of patients studied, variability in temporality of blood sample collection, as well as an incomplete data set for echocardiogram measured LVEF and longer term follow up for cardiac function. A control group of patients not receiving chemotherapy is not available, however the pre-treatment LVEF analysis by 2D echo provide an internal control. Echocardiograms were ordered at the discretion of the attending clinic physician and not all patients had LVEF measured.

## Conclusions

In summary, low levels of RNA virus sequences are detectable in venous blood samples taken from patients with hematological cancers after chemotherapy. Analysis of these detected RNA virus sequences suggest that there is increased detection of RNA virus sequences in blood samples from patients after chemotherapy. Chemotherapeutic immune suppression increases the risk for chemotherapy induced myocarditis and cardiomyopathy with reduced LVEF. A comprehensive, structured study of cancer patients for sequential detection for RNA virus sequences after chemotherapy and correlation with LVEF analysis at predetermined follow up times, as well as an analysis for other markers for cardiac damage and immunosuppression is needed. A structured sequential analysis, as well as a comparison to patients without chemotherapy, will allow identification of opportunistic viral infections and identify potential approaches to prevent or treat viruses identified as risk factors for myocarditis and cardiomyopathy after chemotherapy.

## Clinical Perspectives—Translational Outlook

Further investigation into the role of RNA viruses as a significant underlying etiology for myocarditis and cardiomyopathy after chemotherapy with associated immunosuppression is needed.

Understanding the role of virus induced myocarditis and cardiomyopathy after chemotherapy will allow for further treatment, both preventative through vaccines and for selective anti-viral treatment.

## Data Availability Statement

The original contributions presented in the study are publicly available. This data can be found here: NCBI, BioProject, PRJNA79842.

## Ethics Statement

The studies involving human participants were reviewed and approved by Biodesign Institute, ASU IRB and Department of Medicine, Shands Hospital, University of Florida. The patients/participants provided their written informed consent to participate in this study.

## Author Contributions

KV, ST, RB, MJ, SS-C, CP, and AL designed the experiment and wrote the manuscript. ST, SA, CS, MH, SS-C, and AL managed participant recruitment and were responsible for sample acquisition and preservation. AL, ST, KV, RB, SK, JY, LZ, MJ, PM, and AV analyzed and interpreted the data. All authors contributed to manuscript revision, provided important intellectual contributions, and approved the submitted version.

## Funding

This work was supported by Biodesign, ASU startup funding (AL), American Heart Association (AL), National Institutes of Health (AL), and St Jude Children's Research Hospital (SS-C).

## Conflict of Interest

The authors declare that the research was conducted in the absence of any commercial or financial relationships that could be construed as a potential conflict of interest.

## Publisher's Note

All claims expressed in this article are solely those of the authors and do not necessarily represent those of their affiliated organizations, or those of the publisher, the editors and the reviewers. Any product that may be evaluated in this article, or claim that may be made by its manufacturer, is not guaranteed or endorsed by the publisher.

## Supplementary Material

Supplementary Figure 1 Pavian software illustrations of detected RNA virus reads detected based on Kraken 2 analyses for all individual patient samples.
