# Supplementary figures and images for: RNA Virus Gene Signatures Detected in Patients With Cardiomyopathy After Chemotherapy; A Pilot Study
Source: Front Cardiovasc Med. 2022 Mar 11;9:821162. doi: 10.3389/fcvm.2022.821162 (PMC8962958; doi:10.3389/fcvm.2022.821162)

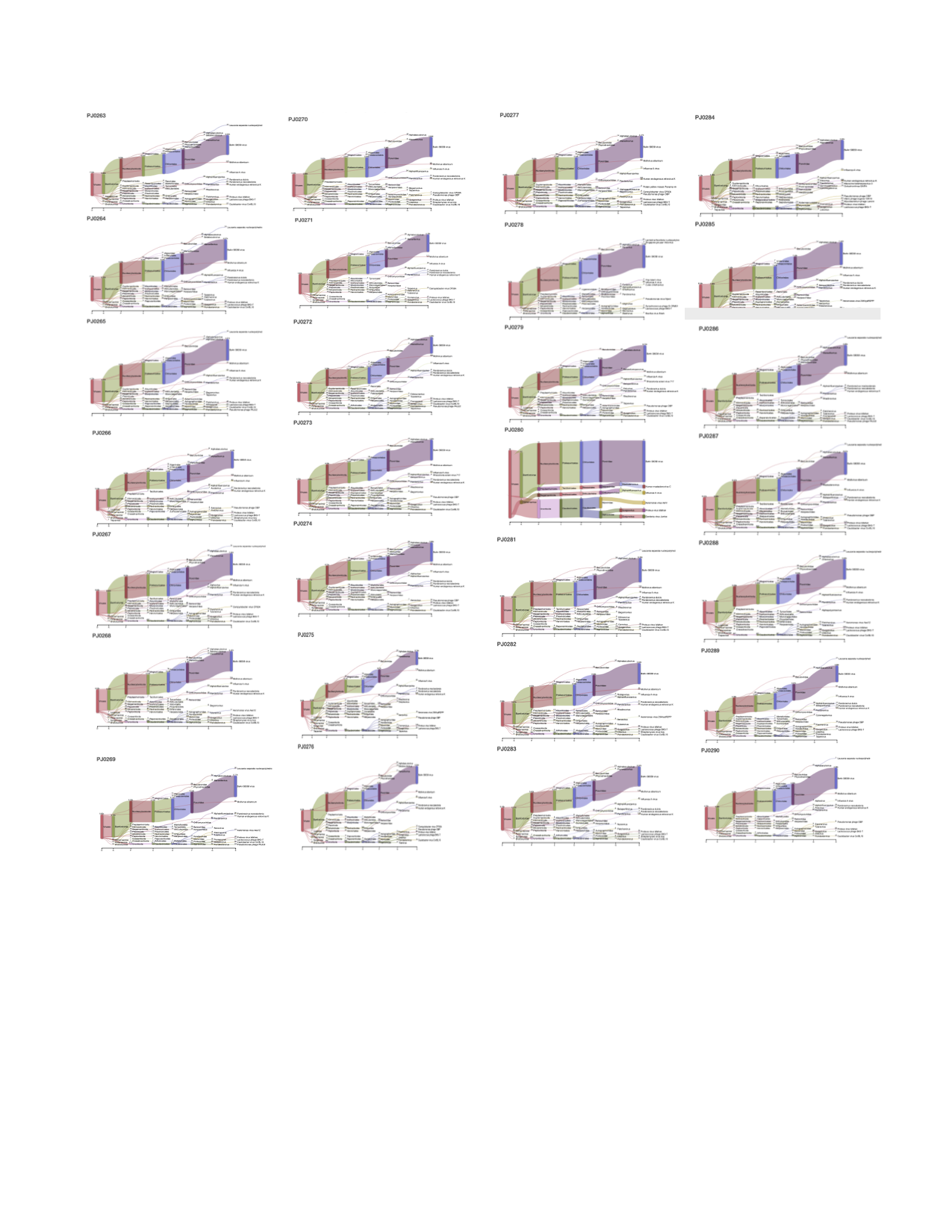

Supplement: Supplementary Figure 1 — Pavian software illustrations of detected RNA virus reads detected based on Kraken 2 analyses for all individual patient samples. [file Image_1.TIFF]
